# Systematic Artifacts in Support Vector Regression-Based Compound Potency Prediction Revealed by Statistical and Activity Landscape Analysis

**DOI:** 10.1371/journal.pone.0119301

**Published:** 2015-03-05

**Authors:** Jenny Balfer, Jürgen Bajorath

**Affiliations:** Department of Life Science Informatics, B-IT, LIMES Program Unit Chemical Biology and Medicinal Chemistry, Rheinische Friedrich-Wilhelms-Universität, Dahlmannstr. 2, D-53113, Bonn, Germany; University of Bologna & Italian Institute of Technology, ITALY

## Abstract

Support vector machines are a popular machine learning method for many classification tasks in biology and chemistry. In addition, the support vector regression (SVR) variant is widely used for numerical property predictions. In chemoinformatics and pharmaceutical research, SVR has become the probably most popular approach for modeling of non-linear structure-activity relationships (SARs) and predicting compound potency values. Herein, we have systematically generated and analyzed SVR prediction models for a variety of compound data sets with different SAR characteristics. Although these SVR models were accurate on the basis of global prediction statistics and not prone to overfitting, they were found to consistently mispredict highly potent compounds. Hence, in regions of local SAR discontinuity, SVR prediction models displayed clear limitations. Compared to observed activity landscapes of compound data sets, landscapes generated on the basis of SVR potency predictions were partly flattened and activity cliff information was lost. Taken together, these findings have implications for practical SVR applications. In particular, prospective SVR-based potency predictions should be considered with caution because artificially low predictions are very likely for highly potent candidate compounds, the most important prediction targets.

## Introduction

Support vector machines (SVMs) are algorithms for supervised machine learning [1] that have become increasingly popular for object classification and ranking in bioinformatics [2,3] and chemoinformatics [4,5], given their often observed high predictive performance compared to other machine learning approaches [5]. The basic idea underlying SVM modeling is to derive classification models by separating positive and negative training data with the largest possible margin. Furthermore, SVMs are often used in combination with kernel functions, which project training sets into feature spaces of higher dimensionality where a linear separation of positive and negative training data might ultimately be feasible. The resulting models are then used to predict test instances.

In addition to classification and ranking, the SVM approach has also been adapted for prediction of numerical property values through support vector regression (SVR) [6,7]. Instead of constructing a hyperplane for classification, SVR derives a function on the basis of training data to predict numerical values. SVR is an intrinsically non-linear prediction approach because it projects data sets characterized by the presence of non-linear structure-property relationships in original feature spaces into higher-dimensional space representations where a linear regression function can be fitted. Accordingly, SVR has been receiving much attention in recent years in the context of quantitative structure-activity relationship analysis (QSAR) [8] to predict activities of bioactive compounds. QSAR has been, and continues to be, the most widely applied computational approach for potency prediction and compound design in medicinal chemistry.

Classical QSAR modeling attempts to predict changes in compound potency that result from small chemical modifications using linear regression models [8]. Therefore, these predictions are typically limited to series of structural analogs in which the assumption of at least approximate linearity of structure-activity relationships (SARs) holds. By contrast, the prediction of potency values of compounds from large and structurally heterogeneous data sets, in which SARs are typically non-linear, fall outside the applicability domain of classical QSAR and require non-linear prediction methods such as neural networks [8] or SVR. In addition to potency prediction [9–11], SVR has also been applied to predict a variety of other compound-associated property values [12–16]. SVR models derived for potency prediction reported in the literature are typically statistically assessed and cross-validated following standard QSAR procedures, i.e., by calculating coefficients of determination to account for the ability of a model to fit the potency values of the training data and predict test data not utilized for model building [8].

In this work, we have carried out an in-depth performance evaluation of SVR models for potency prediction beyond standard statistics. SVR models were derived for a variety of data sets with different SAR characteristics and systematically analyzed for their ability to predict compound potency values and vulnerability to over- or underfitting potency data. Furthermore, SVR model regularization terms were systematically varied to balance model complexity and permitted training errors in different ways. For all data sets, activity landscapes [17] were generated from experimental measurements and compared to landscape representations derived on the basis of SVR predictions. Although SVR models were generally statistically sound due to accurate predictions of many intermediate potency values, the models were affected by underfitting and consistently inaccurate predictions of the most potent compounds, leading to a smoothing effect on modeled activity landscapes and loss of critical SAR information.

## Materials and Methods

### Compound data selection

From the public database ChEMBL [18], release 17, all sets of compounds active against human targets were selected that contained at least 500 molecules. Furthermore, qualifying compounds were required to be experimentally tested in a direct inhibition or binding assay with highest ChEMBL confidence score. Only equilibrium constants (K_i_ values) below 100 μM were considered, hence omitting weakly active compounds and assay-dependent measurements. Multiple K_i_ values available for the same compound were averaged if they fell into the same order of magnitude; otherwise, the compound was discarded. Furthermore, duplicates, known pan-assay interference compounds [19], and other reactive molecules were removed from all data sets using in-house computational filters. On the basis of these stringent selection criteria, 31 compounds sets with activities against diverse targets were obtained for SVR modeling, as summarized in Table 1. All data sets are freely available for download from the public ZENODO platform [20].

**Table 1:** Data overview.

TID	Target name	no. of cpds.	min.pKi	max.pKi	mean pKi
11	Thrombin	654	5.00	12.19	6.78
15	Carbonic anhydrase II	1221	5.00	9.41	6.97
51	Serotonin 1a (5-HT1a) receptor	1342	5.05	10.85	7.74
72	Dopamine D2 receptor	1791	5.00	10.24	6.97
87	Cannabinoid CB1 receptor	1661	5.00	10.10	6.92
100	Norepinephrine transporter	928	5.03	9.66	6.94
107	Serotonin 2a (5-HT2a) receptor	824	5.01	11.00	7.54
108	Serotonin 2c (5-HT2c) receptor	577	5.00	9.70	7.01
114	Adenosine A1 receptor	1911	5.01	10.52	6.61
121	Serotonin transporter	1229	5.02	10.89	7.44
129	Mu opioid receptor	1504	5.01	11.80	7.45
130	Dopamine D3 receptor	1142	5.05	10.05	7.37
136	Delta opioid receptor	1203	5.01	10.60	7.19
137	Kappa opioid receptor	1399	5.02	11.52	7.50
138	Nociceptin receptor	642	5.04	10.70	7.84
155	Dopamine transporter	745	5.04	9.80	6.70
165	HERG	701	5.00	9.26	6.14
176	Purinergic receptor P2Y12	536	5.36	9.40	7.81
194	Coagulation factor X	1129	5.02	11.40	8.05
252	Adenosine A2a receptor	2189	5.01	11.09	6.91
259	Cannabinoid CB2 receptor	1841	5.00	10.40	7.17
278	Adenosine A2b receptor	856	5.05	9.80	7.31
280	Adenosine A3 receptor	1766	5.02	10.56	7.20
10142	Melanocortin receptor 4	1199	5.01	9.40	6.96
10193	Carbonic anhydrase I	1134	5.00	10.68	6.33
10280	Histamine H3 receptor	1861	5.04	10.50	7.95
10627	Serotonin 6 (5-HT6) receptor	1157	5.06	10.30	7.76
11290	Histamine H4 receptor	596	5.04	10.40	7.08
12209	Carbonic anhydrase XII	717	5.00	9.52	7.24
12952	Carbonic anhydrase IX	1033	5.01	9.92	7.07
19905	Melanin-concentrating hormone receptor 1	701	5.03	9.77	7.42

For all 31 data sets used for SVR modeling, the ChEMBL target ID (TID), target name, number of compounds, and the minimum, maximum, and mean pK_i_ values are reported.

### Molecular representations

For all test compounds, two fingerprints were calculated as descriptors including molecular access system (MACCS) keys [21] and the extended connectivity fingerprint with bond diameter 4 (ECFP4) [22]. MACCS is a fixed-length fingerprint consisting of 166 pre-defined substructural patterns and ECFP4 a topological atom environment fingerprint of higher chemical resolution. ECFP generates all possible atom environments up to a layer of four bonds around each atom. The resulting atom environments represent a feature set of data-specific size. Both fingerprint representations were calculated using in-house implementations based upon OpenEye’s OEChem toolkit [23].

### SAR information content

The SAR characteristics of the 31 compound data sets used for SVR modeling were quantitatively described using the continuity and discontinuity score components of the SAR Index (SARI) [24]. For all data sets, initial scores were calculated as follows:

$${\text{cont}}_{\text{raw}}=1-\frac{\sum_{i>j}w_{ij}Tc(i,j)}{\sum_{i>j}w_{ij}}$$ (1)

$${\text{disc}}_{\text{raw}}=\frac{\sum_{\left\{i,j|Tc(i,j)\geq t,i>j\right\}}\left|pot(i)-pot(j)\right|Tc(i,j)}{\left|\left\{i,j|Tc(i,j)\geq t,i>j\right\}\right|}$$ (2)

Here, the *Tc(i*,*j)* is the Tanimoto coefficient [25,26] of ligands *i* and *j*, *w*_*ij*_ is a weight defined as$\frac{pot(i)pot(j)}{1+\left|pot(i)-pot(j)\right|}$, and *pot(i)* is the potency of compound *i* as pKi value [24]. For the discontinuity score, we used corresponding Tc similarity threshold values of *t = 0*.*85* for MACCS and *t = 0*.*56* for ECFP4 [27]. Raw scores were then converted into Z-scores and normalized using the cumulative normal distribution. For this purpose, 120 compound data sets containing at least 100 compounds each were extracted from ChEMBL on the basis of the selection criteria specified above and used as an external reference panel.

### Activity landscapes

In chemoinformatics and medicinal chemistry, activity landscapes of compound data sets are generally defined as graphical representations that integrate molecular similarity and activity relationships between compounds [17]. In addition to numerical SAR characterization, three-dimensional (3D) activity landscape views [28] were calculated for all compound data sets subjected to SVR modeling. The 3D activity landscape representation was calculated as described previously based upon a two-dimensional (2D) projection of all pairwise Tc values for a compound set using multi-dimensional scaling [28]. This projection provides a 2D similarity map that is then complemented by an activity surface interpolated from compound potency values. The potency surface is then added to the projection, yielding a 3D activity landscape representation reminiscent of geographical landscape views.

### Support vector regression modeling

#### Support vector regression theory

Support vector regression (SVR) is a supervised machine learning method for the prediction of numerical target values [6,7]. For training, labeled examples are mapped into a descriptor space and a function of the form

f(x)=〈w,x〉+b (3)

is derived that best predicts the target values for the examples *x*. The parameters *w*,*b* are derived via the following optimization:

$$\begin{array}{l} \min\frac{1}{2}{\left\|w\right\|}^{2}+C\sum_{i=1}^{n}\left(\xi_{i}+\xi_{i}^{*}\right)\\ \text{subject to }\{\begin{array}{ll} y_{i}-\langle w,x_{i}\rangle -b & \leq \epsilon +\text{​}\xi_{i}\\ \langle w,x_{i}\rangle +b-y_{i} & \leq \epsilon +\text{​}\xi_{i}^{*}\\ \xi_{i},\xi_{i}^{*} & \geq 0 \end{array} \end{array}$$ (4)

This concept is derived from support vector machines (SVMs), which were introduced for binary classification tasks. While SVMs attempt to maximize the margin between two classes, SVR derives a so-called *ε-insensitive tube* around the target values [7]. The width of this tube, ε, provides the amount of permitted error, i.e., target values that are mispredicted by less than ε are not penalized by the optimization. Training examples that are predicted with a deviation of more than ε from their true target value fall outside the tube and are called *support vectors*.

Furthermore, ξ_i_ ξ_i_* in formula (4) are sets of non-negative *slack variables* permitting a certain violation of the ε-tube’s bounds [29]. The *regularization term C* balances the cost of a complex model with the cost of training errors: if *C* is large, training errors are strongly penalized and the derived model is highly complex, thus entailing a risk of data *overfitting*. In contrast, if the regularization term is small, low-complexity models are favored at the risk of *underfitting*. In the linear case, the vector *w* can be written as a weighted combination of the support vectors:

$$w=\sum_{i}\left(\alpha_{i}-\alpha_{i}^{*}\right)x_{i}$$ (5)

Hence, the prediction function can be expressed as:

$$f(x)=\sum_{i}(\alpha_{i}-\alpha_{i}^{*})\langle x_{i},x\rangle +b$$ (6)

Importantly, if a linear regression modeling of the training data in the given space is not possible, the scalar product 〈·,·〉 can be replaced by a kernel function *K(u*,*v)* to project the data into higher dimensional space in which a linear separation becomes feasible, in analogy to SVMs. This procedure is generally referred to as the *kernel trick* [30]. If the kernel trick is applied, the weight vector *w* can no longer be directly expressed and the prediction function changes to:

$$f(x)=\sum_{i}\left(\alpha_{i}-\alpha_{i}^{*})K(x_{i},x\right)+b$$ (7)

In analogy to Tc-based similarity calculations, the *Tanimoto kernel* [31] is often used as a kernel function for compound potency prediction:

$$K(u,v)=\frac{\langle u,v\rangle }{\langle u,u\rangle +\langle v,v\rangle -\langle u,v\rangle }$$ (8)

#### Performance analysis

To assess the overall performance of the SVR modeling, we have calculated absolute errors, mean absolute errors, and R^2^ values. According to equation (9), the absolute error was defined as given by the objective function:

$$\sum_{i=1}^{n}\left(\xi_{i}+\xi_{i}^{*}\right)=\overset{n}{\underset{i=1}{\sum }}\max\left(0,\left|y_{i}-f(x_{i})\right|-\epsilon \right)$$ (9)

While this is not the standard formula for the absolute error, it reflects the training of the models with a certain amount of permitted deviation. This value was used to determine the best regularization term for each model, provided by the value for *C* that resulted in the lowest absolute error on the test set.

The mean absolute error is given as the absolute error divided by *n*, the number of compounds. Furthermore, coefficients of determination (R^2^) values were computed. R^2^ quantifies how much of the data variance can be explained by the model.

### Calculation set-up

For each data set, 55 different SVR models were trained with *C* varying in {1, 2, 3…, 50, 100, 250, 500, 1000}. Hence, these models covered a wide range of regularization parameters enabling the analysis of potential under- and overfitting effects. All SVR models were derived using the freely available Python implementation scikit-learn [32] with parameter setting ε = 0.1. We have kept the parameter ε constant instead of optimizing it considering the nature of the data. Because target values were pKi values (which are well-defined) setting ε to values smaller than 0.1 would be beyond experimental detection limits. Moreover, variations of larger values are also not meaningful because deviations of close to one order of magnitude or more are biologically relevant and should not be treated as allowed deviations. For each of the 55 different *C* settings, 10 models were built with randomly chosen training and test sets, each comprising of 50% of the data (yielding a total of 550 models per set). In each case, prediction performance was averaged over all 10 independently derived models.

## Results and Discussion

### Regularization analysis

Initially, the effects of regularization term variations on SVR model performance were evaluated in detail. Therefore, the mean training and test errors over all individual trials were determined for each setting of *C*. Because this regularization parameter balances model complexity and permitted training errors, its variation makes it possible to elucidate the tendency of over- or underfitting of the models. For small values of *C*, both training and test errors are typically high, which then provides a clear indication of underfitting. Increasing values of *C* should then lead to a decrease in training and test error, indicating a better fit of a model. The application of increasingly large values of *C* values typically leads to increasing test errors in the presence of constant or further decreased training errors, which indicates overfitting of a model.

**Fig 1:**
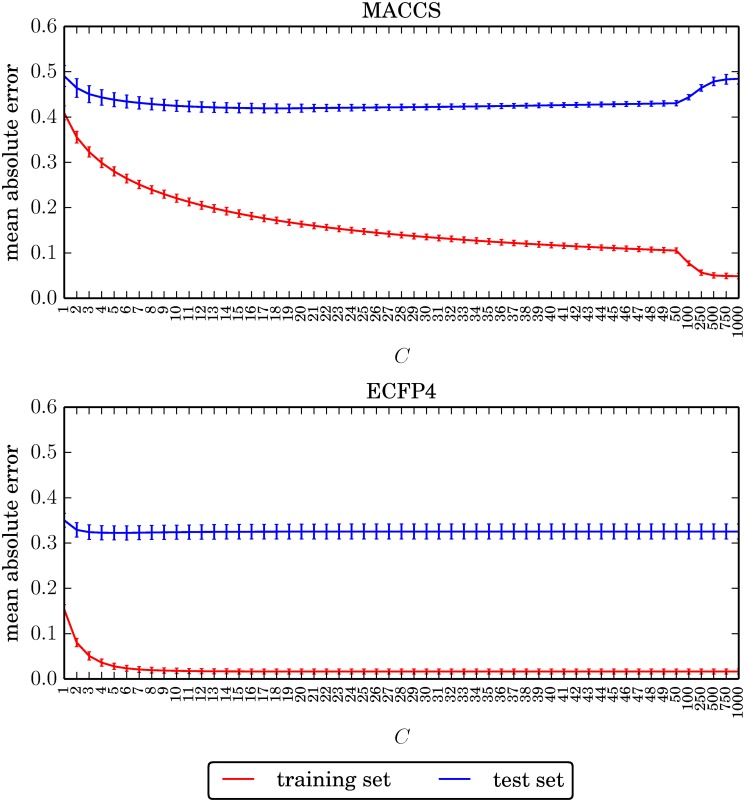
Exemplary regularization profile. For each value of the regularization term *C*, the absolute training and test error averaged over all trials is reported for data set TID 10280. Error bars give the standard deviations. Regularization values are plotted evenly on the x axis, regardless of their magnitude.

On the basis of these general considerations, the value of *C* yielding the smallest test error was selected as the preferred regularization term for each data set / fingerprint combination. Table 2 reports the preferred values for all data sets and fingerprints. The performance of the resulting models is further discussed in the following. It should be noted that in benchmark studies, parameters are typically evaluated on an external validation set, while performance values are reported on a distinct test set. However, in this study, we have not aimed to benchmark SVR models and deliberately introduced a positive bias towards model performance to emphasize the consistently observed failure in correctly predicting the most important compounds, as further discussed below. In our regularization analysis, consistent trends were observed across all data sets. Fig. 1 reports the mean training and test errors under regularization term variation for an exemplary data set (melanocortin receptor 4 antagonists, TID 10142), which mirrors the observed trends. First, both training and test errors were consistently higher for MACCS- than for ECFP4-based models. As a consequence, effects of underfitting and, in part, overfitting depending on the choice of *C* were clearly observed for MACCS but to a much lesser extent, if at all, for ECFP4. The training error of the ECFP4 model reached its minimum of 0.0165 already at *C = 19*, while the training error of the MACCS model continued to steadily decrease for increasing values of *C*. However, for both models, the test error essentially remained constant over most regularization term settings. Only for the highest *C* values, an overfitting effect became apparent for MACCS. This was in contrast to the ECFP4 model that did not reveal an apparent overfit at any point. Moreover, regardless of the data set and molecular representation used, the difference between training and test errors was consistently large. These results revealed that the SVR models in combination with a suitable high-dimensional representation (i.e., ECFP4 instead of MACCS) did not display a notable tendency of overfitting, which often severely affects QSAR modeling [8]. By contrast, underfitting of SVR models was observed for low regularization terms.

**Table 2:** Best regularization values.

TID	MACCS	ECFP4
11	23	8
15	10	3
51	17	3
72	7	5
87	8	3
100	6	2
107	7	3
108	7	3
114	11	4
121	9	4
129	16	7
130	9	7
136	12	4
137	14	3
138	6	3
155	7	3
165	9	3
176	12	2
194	11	5
252	14	4
259	9	4
278	9	3
280	27	6
10142	18	6
10193	11	2
10280	14	4
10627	12	3
11290	16	5
12209	16	5
12952	14	4
19905	5	6

Reported are the values of the regularization parameter *C* yielding the lowest test error for each data set and fingerprint representation.

### Regression performance

**Fig 2:**
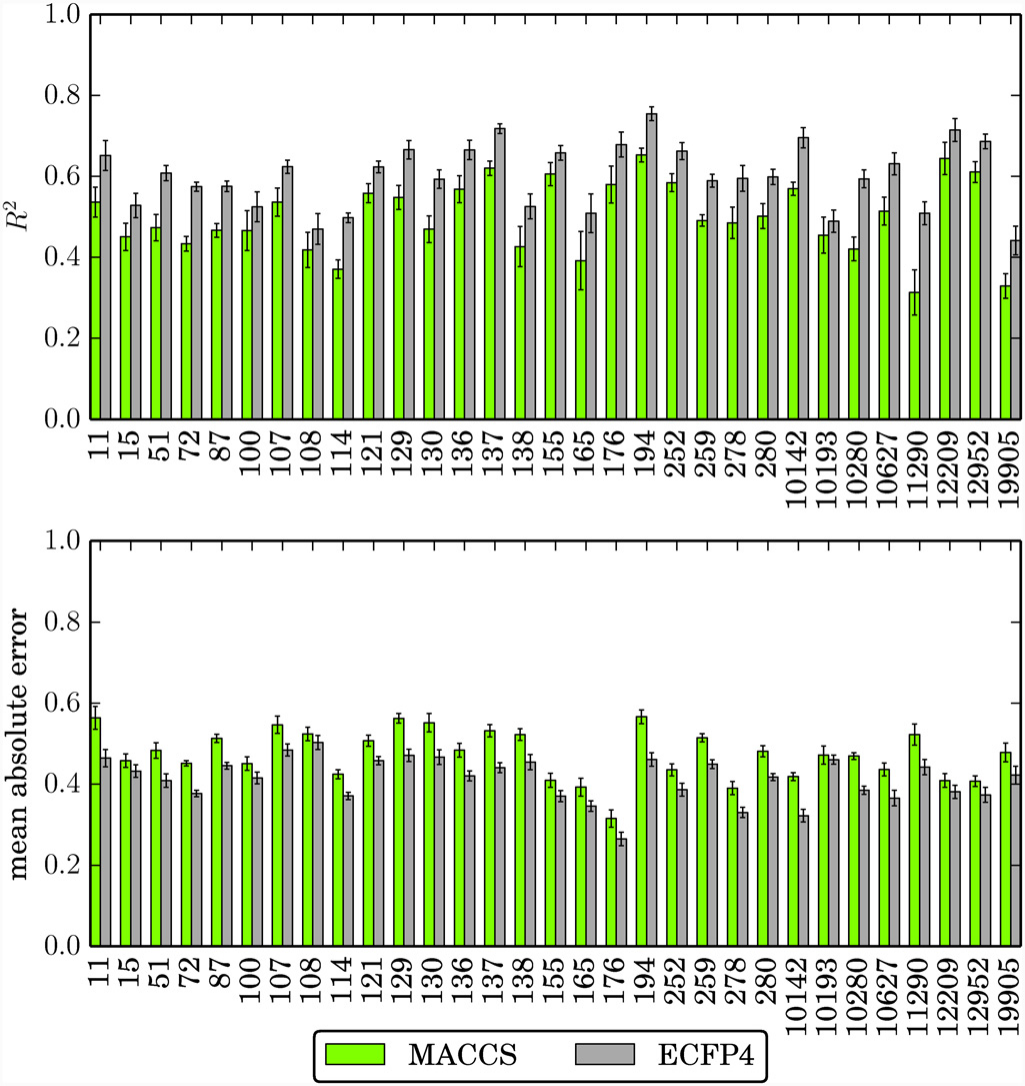
Global regression performance. Reported are the mean R^2^ and mean absolute error values for each data set and fingerprint, determined on the test set. Error bars give the standard deviations.

We next determined global regression accuracy of the SVR models on the basis of R^2^ values and mean absolute errors calculated on the test set. Therefore, for each data set, the overall best performing model identifed under systematic variation of regularization parameter *C* was selected. R^2^ values and mean absolute errors of these models are shown in Fig. 2. For all data sets, the performance using MACCS was lower than for the higher-resolution topological ECFP4 fingerprint. MACCS-based models also required generally higher regularization term values than ECFP4-based models (Table 2). Overall, R^2^ values ranged from 0.31 and 0.65 (MACCS) and from 0.44 and 0.75 (ECFP4). Hence, these values were of moderate magnitude. However, mean prediction errors were generally low and fell into the pK_i_ value intervals [0.32, 0.57] and [0.26, 0.50] for MACCS and ECFP4, respectively. Thus, compound potencies were generally predicted well within an order of magnitude, which is considered encouraging accuracy from a QSAR perspective. Of course, the most interesting test compounds for prediction across large data sets were those with high potency. However, prediction results for individual compounds cannot be rationalized on the basis of average errors. Therefore, alternative measures were applied to evaluate the quality of the SVR predictions for potent compounds, as discussed in the following.

### SAR characteristics and predictive performance

SAR continuity and discontinuity scores were calculated for all data sets to characterize their global SAR information and relate these SAR characteristics to SVR model performance. High discontinuity scores indicate the presence of many structurally similar compounds with large potency variations, whereas high continuity scores account for the presence of structurally similar or dissimilar compounds with small to moderate variations in potency [24]. Activity cliffs, which consist of pairs or groups of structurally analogous compounds with largest differences in potency, represent the extreme form of SAR discontinuity in a data set [33]. They also represent the most prominent and informative features of activity landscapes [17]. In large and structurally heterogeneous data sets, as investigated herein, continuous and discontinuous SAR environments typically coexist and determine the global SAR phenotype [17,24].

**Fig 3:**
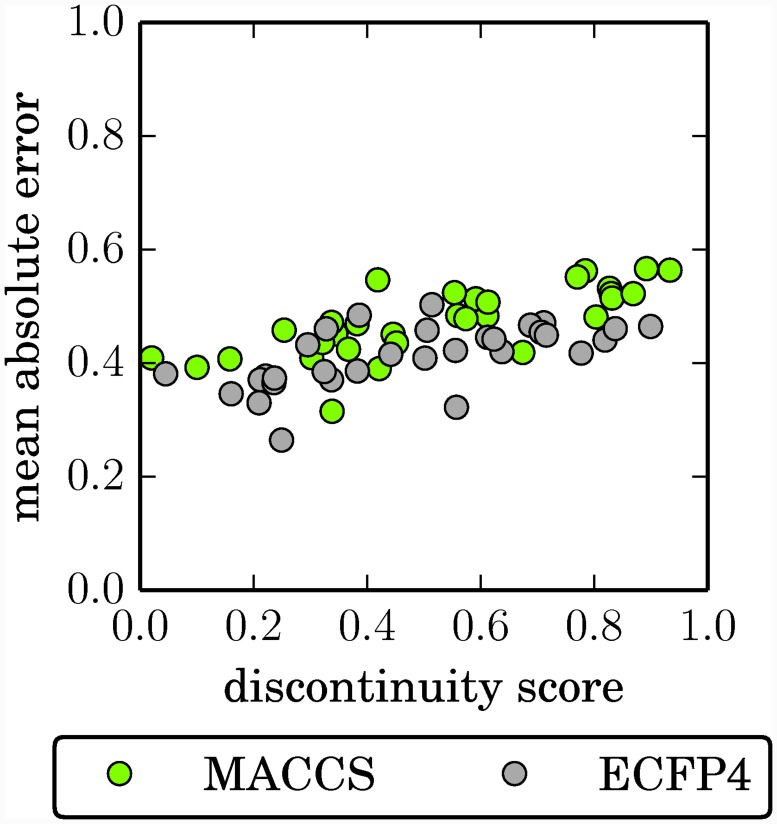
SAR discontinuity vs. SVR error. For each data set and fingerprint, the global discontinuity score is plotted against the mean absolute error of the SVR model, determined on the test set. Prediction errors display the tendency to increase with increasing discontinuity scores.

To evaluate if differences in SAR characteristics influenced the fit of SVR models, the correlation of continuity / discontinuity scores and test errors was assessed. To render test errors independent of data set size, the mean error over all test compounds was determined and the Pearson product-moment correlation coefficient between SAR scores and the mean error was calculated. In addition, a two-tailed p-value was determined to assess the statistical significance of correlation effects. These calculations revealed no notable correlation between the SAR continuity score and the prediction error, but a statistically significant correlation (p < 0.01) between the discontinuity score and the prediction error. The corresponding Pearson correlation coefficient was 0.75 for MACCS and 0.61 for ECFP4, which indicated the presence of a moderate positive correlation between the discontinuity scores and the mean test error. Thus, the more discontinuous a data set was at the global SAR level, the higher was the prediction error. In Fig. 3, the discontinuity scores of all data sets are plotted against their mean test errors, which reflects this trend.

### SVR-based reproducibility of SAR characteristics

#### Discontinuity scores based on predicted potency values

To further evaluate the findings discussed above, we recalculated continuity and discontinuity scores on the basis of potency values predicted for all training and test set compounds. The *predicted training set* continuity / discontinuity was calculated on the basis of potency predictions for training set compounds and provides an estimate of the influence of the training error and model fit on SAR characteristics. In contrast, the *predicted test set* continuity / discontinuity was calculated based on test set potency predictions and reflects the generalization ability of the model and its influence on prediction accuracy. Furthermore, in the following, *observed* continuity / discontinuity refers to the respective scores calculated on the basis of experimental potency values. For score calculations on the basis of predicted values, the mean predicted potency of each test and training set compound was determined over all trials. The few compounds that never occurred in any of the randomly selected training or test sets were assigned their observed potency value.

**Fig 4:**
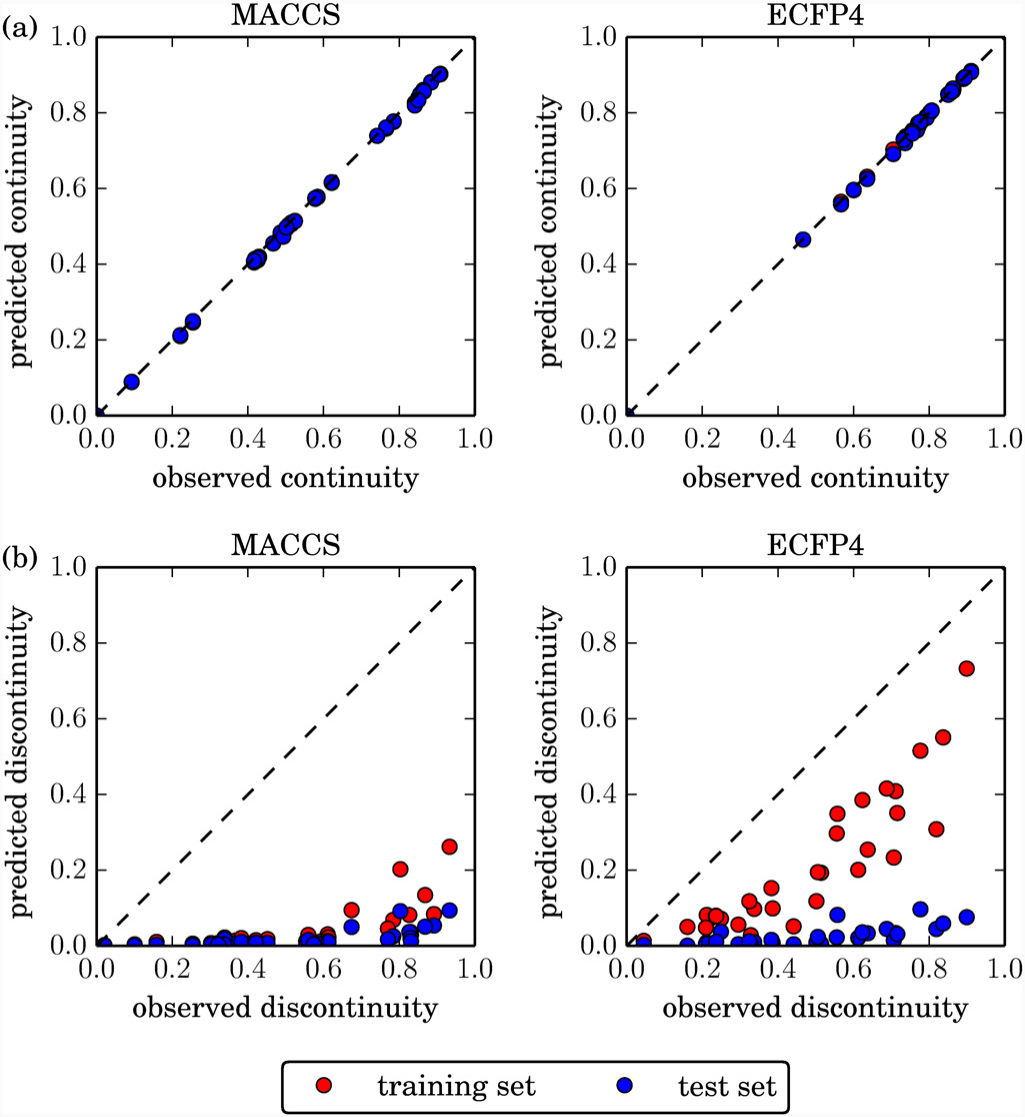
Observed vs. predicted SAR characteristics. The observed global (a) continuity and (b) discontinuity score of each data set and fingerprint is plotted against the predicted training set (red) and test set (blue) value (blue and red data points are placed in the fore- and background, respectively).

Fig. 4 reports the comparison of predicted and observed SAR scores. Observed continuity scores of all data sets were almost perfectly reproduced (Fig. 4a). By contrast, predicted discontinuity scores were consistently and significantly lower than observed scores (Fig. 4b). For both MACCS and ECFP4, predicted test set discontinuity scores were very low (mostly below 0.1). The predicted training set scores were only slightly higher for MACCS, but substantially higher for ECFP4. Nevertheless, even for ECFP4, scores for training set compounds were consistently underpredicted.

#### Limited generalization ability in discontinuous SAR regions

The comparison of predicted training and test set continuity scores showed that the SVR models fit continuous training data subsets very well and were generalizable for yielding high-quality potency prediction on continuous test data. In contrast, the comparison of predicted training and test set discontinuity scores indicated that only the ECFP4-based models partly fit discontinuous training data, but did not generalize well for potency predictions on test compounds in discontinuous SAR environments. Thus, SAR characteristics substantially influenced the quality of SVR-based predictions. Potency patterns in discontinuous SAR regions were generally difficult to predict.

#### Influence of regularization on discontinuity scores

**Fig 5:**
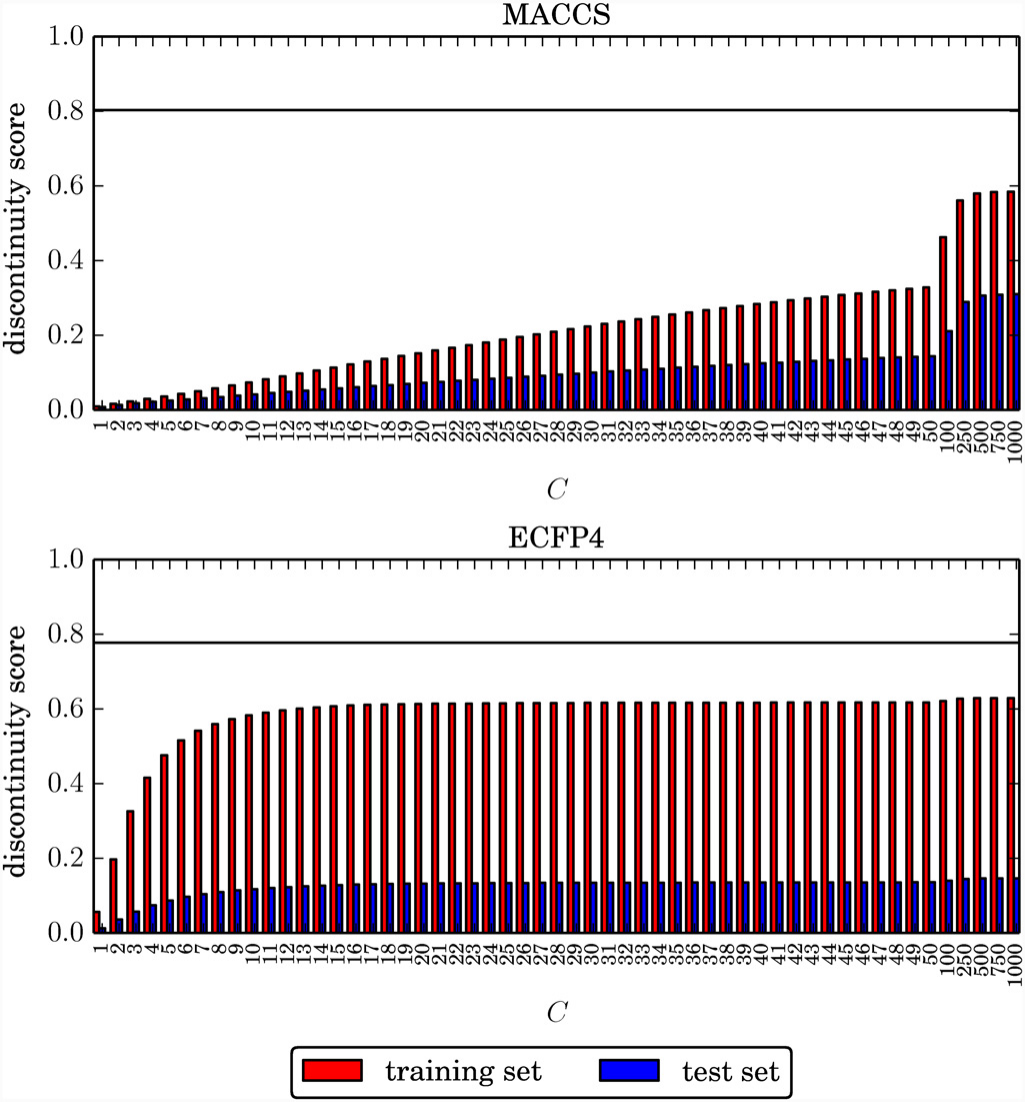
Exemplary discontinuity score profile. For each value of the regularization term *C*, the discontinuity score resulting from the predicted training and test set potency values is reported for data set TID 280. The black line denotes the observed discontinuity score of the data set. Regularization values are evenly spaced on the horizontal axis, regardless of their magnitude.

In light of these findings, we also examined how the choice of the regularization term *C* influenced the magnitude of discontinuity scores based upon predicted potency values. Fig. 5 reports the discontinuity scores calculated on the basis of predicted test and training set potencies plotted against the regularization term for an exemplary data set (adenosine A3 receptor antagonists, TID 280). The horizontal black line denotes the observed discontinuity score calculated from the experimental potency values. Although observed discontinuity scores varied for all data sets, the trends in Fig. 5 were consistently detected. Discontinuity scores calculated with MACCS based upon predicted potencies gradually increased with increasing *C* values (consistent with the observation that training errors decreased with increasing *C*, as discussed above). In this case, the largest regularization terms yielded highest (albeit still substantially underpredicted) discontinuity scores. By contrast, for ECFP4, the discontinuity scores rapidly increased for small C values and then essentially remained constant, with much higher scores achieved on the basis of predicted training set than test set potency values.

#### Plausible rationale for potency prediction errors

Taking into consideration that the regularization term balances SVR model complexity with the amount of permitted training errors, we reasoned that the consistent underestimation of discontinuity scores might result from incorrectly predicted potency values in activity cliff regions, for the following reasons: Activity cliffs are generally rare in compound data sets involving on average only ~20% of active compounds [33]. Moreover, because activity cliffs consist of pairs of structurally analogous compounds with largest potency difference in a data set, only a small percentage of highly potent compounds (at most ~10%, but in practice often less [33]) is responsible for their formation. If training errors are permitted for these highly potent compounds, the SVR algorithm is expected to produce an easy to derive, low-complexity model yielding overall accurate predictions for many active compounds in low or intermediate potency ranges. This hypothesis is fully consistent with the regularization profile of ECFP4 reported in Fig. 5, which showed that a stable prediction model was obtained for small values of C, permitting training errors and a limited underfit of the model for the benefit of enabling low-complexity predictions.

### SVR modeling effects on activity landscapes

**Fig 6:**
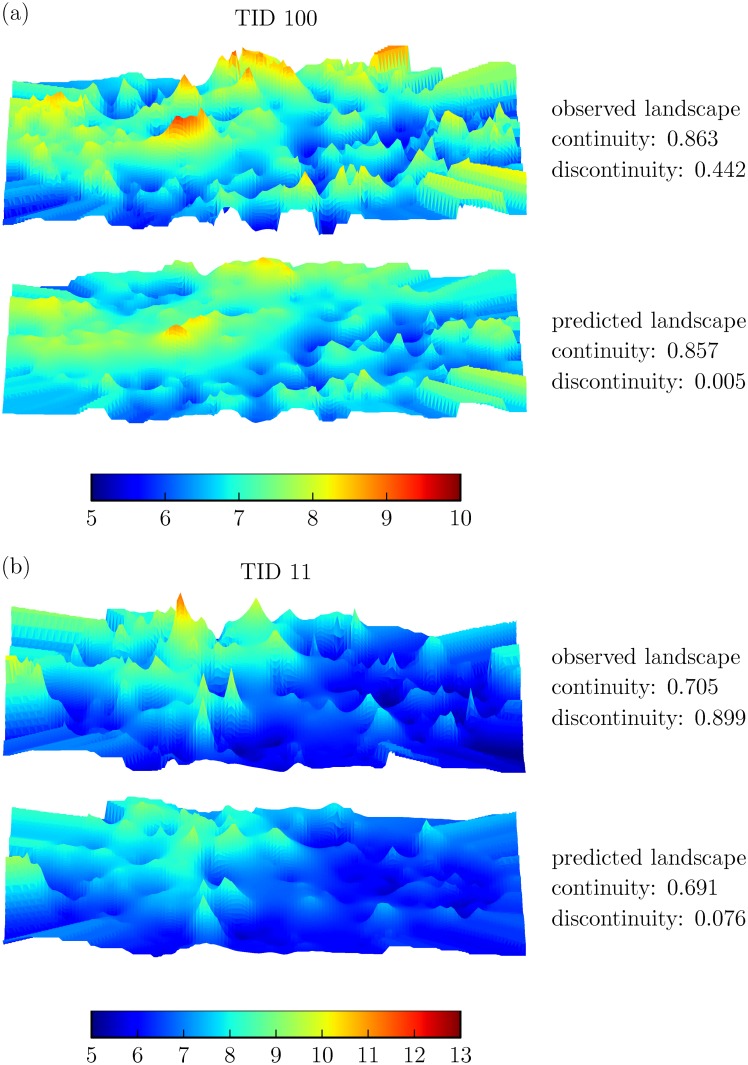
Observed and predicted activity landscapes. For two representative data sets, observed and predicted 3D activity landscape representations are compared and corresponding continuity and discontinuity scores are reported. Landscape surface elevation correlates with increasing potency. The activity landscape views are represented applying a continuous spectrum from blue to red spanning a potency range of (a) 5 to 10 pK_i_ (TID 100) and (b) 5 to 13 pK_i_ (TID 11). Hence, the positions of the most potent compounds in the landscapes are colored red.

In order to evaluate the hypothesis formulated above, we generated 3D activity landscapes [28] for our data sets on the basis of experimental potency values and compared them to corresponding landscapes generated on the basis of potency values predicted for the test sets. Since essentially all data set compounds with only few exceptions occurred multiple times (or at least once) in training and test sets (see Methods), these landscape views had conserved topology and could thus be directly compared. Fig. 6 shows ECFP4-based activity landscape comparisons for two exemplary data sets that illustrate consistently detected effects. These data sets and their observed landscapes were characterized by medium to high degrees of discontinuity (resulting from pairwise potency relationship contributions of similar data set compounds). *Rugged regions* in the original landscapes delineate centers of SAR discontinuity where yellow-to-red *peaks* represent most potent data set compounds involved in the formation of activity cliffs with weakly potent structural analogs. In addition, blue *valleys* or blue-to-yellow *plateaus* in these landscapes delineate continuous SAR regions. Comparing the observed and predicted activity landscapes, major “smoothing” effects became apparent: In the predicted landscapes, the most prominent peaks in the original landscapes disappeared and rugged regions were flattened, consistent with the underprediction of SAR discontinuity. Artificial smoothing of activity landscapes directly resulted from incorrect predictions for the small proportion of highly potent compounds. For these compounds, much too low potency values were predicted by the SVR models and, consequently, the activity cliffs they formed were no longer detectable. Thus, consistent with conclusions drawn from SVR vs. SAR analysis, mispredictions of the most potent dataset compounds were the price to pay for obtaining statistically sound SVR models that yielded accurate predictions for the weakly to moderately potent data set compounds.

## Conclusions

In this study, we have analyzed in detail the use of SVR models for compound potency prediction, which represents an increasingly popular QSAR strategy. A major attraction of the SVR approach and other kernel methods are their principal ability to account for non-linear SARs, which sets them apart from classical QSAR methods and enables potency predictions for structurally heterogeneous data sets on a large scale. In order to better understand intrinsic features, opportunities, and limitations of these SVR models, we have systematically analyzed general regression metrics (such as coefficients of determination and error values), model regularization, SAR characteristics, and observed vs. predicted activity landscapes. On the basis of our analysis, a detailed picture of SVR model performance has been obtained, providing a number of implications for practical applications.

For the wide spectrum of high-quality compound data sets with different SAR characteristics we investigated, SVR models with overall low prediction errors were obtained, without subjective intervention. For the majority of compounds across all data sets, potency values were correctly predicted within an order of magnitude, which is in accord with state-of-the-art QSAR standards and within the range of experimental assay variations. These findings provide general support for SVR modeling, consistent with a number of previous studies. We also found that SVR model performance was substantially influenced by the use of alternative molecular representations, which is a known conundrum of machine learning applications in chemoinformatics.

However, our detailed investigation of SVR model performance also yielded a number of new insights, pointing at critical issues that should merit careful consideration. For example, on the basis of regularization parameter analysis, SVR models were robust against data overfitting (consistently stable models were obtained for ECFP4), but generally vulnerable to underfitting. Furthermore, prediction errors were statistically correlated with increasing global SAR discontinuity of compound data sets. In light of these observations, we carefully examined SVR predictions and identified systematic errors in the prediction of highly potent compounds. Even with proper model regularization and a suitable molecular representation such as ECFP4, only highly potent *training set* compounds could be predicted approaching acceptable accuracy (cf. Fig. 5; only the discontinuity scores calculated on predicted training set potencies approach the true data set discontinuity). By contrast, the SVR models lacked the generalization ability to extrapolate from training data to new discontinuous test data and essentially failed to correctly predict high potency values for test compounds. These findings were rationalized by the intrinsic feature of the SVR algorithm to strive for a balance between acceptable training error margins and model complexity. Because highly potent compounds typically represent only a small proportion of a data set, prediction errors can be algorithmically tolerated in these instances to derive a model that is of limited computational complexity but yields sufficiently accurate predictions for the majority of data set compounds. However, for QSAR applications, systematic errors in predicting highly potent compounds are a severe limitation, because such compounds represent prime prediction targets.

When comparing activity landscapes of data sets based upon experimental potency measurements with landscapes generated on the basis of SVR potency predictions, we found that prominent activity cliffs were eliminated by consistently predicting artificially low potency values for highly potent cliff compounds. Thus, SVR-based prediction of activity landscapes resulted in a substantial loss of SAR information. This also meant that the SVM paradigm of nonlinearity, albeit enabling meaningful predictions for structurally heterogeneous data sets as a whole, did not apply to modeling regions of high local SAR discontinuity formed by structural analogs with large potency variations, indicating a principal limitation of the approach.

In summary, our analysis has revealed that care must be taken when utilizing SVR for SAR/QSAR applications, despite promising SVR potency prediction statistics for many different compound data sets. For practical applications, the consistently incorrect prediction of highly potent compounds identified in our analysis represents a likely problem, because most attractive (highly potent) candidate compounds might be missed. However, these issues also present attractive opportunities for future method development, for example, the design of algorithmic SVR variants that would penalize prediction errors in most attractive property ranges.
